# Supplementary material for: Robustness and innovation in synthetic genotype networks
Source: Nat Commun. 2023 Apr 28;14:2454. doi: 10.1038/s41467-023-38033-3 (PMC10147661; doi:10.1038/s41467-023-38033-3)
Supplement: Supplementary file 2 — Reporting Summary [file 41467_2023_38033_MOESM2_ESM.pdf]

nature portfolio

Corresponding author(s): Yolanda Schaerli, Joerg Stelling

Last updated by author(s): Mar 29, 2023

Reporting Summary

Nature Portfolio wishes to improve the reproducibility of the work that we publish. This form provides structure for consistency and transparency in reporting. For further information on Nature Portfolio policies, see our Editorial Policies and the Editorial Policy Checklist.

Statistics

For all statistical analyses, confirm that the following items are present in the figure legend, table legend, main text, or Methods section.

n/a	Confirmed	
☐	☒	The exact sample size (*n*) for each experimental group/condition, given as a discrete number and unit of measurement
☐	☒	A statement on whether measurements were taken from distinct samples or whether the same sample was measured repeatedly
☒	☐	The statistical test(s) used AND whether they are one- or two-sided<br>*Only common tests should be described solely by name; describe more complex techniques in the Methods section.*
☒	☐	A description of all covariates tested
☒	☐	A description of any assumptions or corrections, such as tests of normality and adjustment for multiple comparisons
☐	☒	A full description of the statistical parameters including central tendency (e.g. means) or other basic estimates (e.g. regression coefficient) AND variation (e.g. standard deviation) or associated estimates of uncertainty (e.g. confidence intervals)
☒	☐	For null hypothesis testing, the test statistic (e.g. *F*, *t*, *r*) with confidence intervals, effect sizes, degrees of freedom and *P* value noted<br>*Give P values as exact values whenever suitable.*
☒	☐	For Bayesian analysis, information on the choice of priors and Markov chain Monte Carlo settings
☒	☐	For hierarchical and complex designs, identification of the appropriate level for tests and full reporting of outcomes
☒	☐	Estimates of effect sizes (e.g. Cohen's *d*, Pearson's *r*), indicating how they were calculated

Our web collection on statistics for biologists contains articles on many of the points above.

Software and code

Policy information about availability of computer code

Data collection	Gen5 3.04, Leica Application Suite X 3.4.2.18368
Data analysis	RStudio 1.0.143 (running R 3.4.0), Fiji - ImageJ 2.0.0<br>Code including all model related analysis is available here: https://doi.org/10.3929/ethz-b-000604092<br>Software dependencies are mentioned in the respective README.md. Specifically we used:<br>MATLAB version 2018b and the corresponding toolboxes: MEIGO, IQMtools V1.2.2.2, combinatory, colorBrewer, cbrewer, Violinplot-Matlab<br>Rstudio 2021.09.2 Build 382 and the corresponding package: igraph v1.3.1.<br>Each one of the libraries is accompanied by their respective license and they are provided in the aforementioned link.

For manuscripts utilizing custom algorithms or software that are central to the research but not yet described in published literature, software must be made available to editors and reviewers. We strongly encourage code deposition in a community repository (e.g. GitHub). See the Nature Portfolio guidelines for submitting code & software for further information.

Data

Policy information about availability of data

All manuscripts must include a data availability statement. This statement should provide the following information, where applicable:

- Accession codes, unique identifiers, or web links for publicly available datasets
- A description of any restrictions on data availability
- For clinical datasets or third party data, please ensure that the statement adheres to our policy

The source data underlying Fig. 2, 3b, 3c, 4f, 6c, and 7a, and Supplementary Fig. 1 are provided as a Source Data file (Data S3).
The plasmids used in this study are listed in Supplementary Table 1 and their annotated sequences are provided (Data S2).
The source data and code for generating the model related figures (Fig. 3b, 3d, 4a, 4b, 4c, 4d, 4e, 5a, 5b, 6b, 7b, 7c and Supplementary Figures 2, 3, 4 and 5) are provided here https://doi.org/10.3929/ethz-b-000604092. Model parameters and related assumptions are additionally provided as Data S1.
For comparison of promoter efficiencies we used the data available in the following articles: 1) Anderson, J. et al. BglBricks: A flexible standard for biological part assembly. J. Biol. Eng. 4, 1 (2010), 2) Davis, J.H., Rubin, A.J. & Sauer, R.T. Design, construction and characterization of a set of insulated bacterial promoters. Nucleic Acids Res. 39, 1131-1141 (2011), 3) Kelly, J.R. et al. Measuring the activity of BioBrick promoters using an in vivo reference standard. J. Biol. Eng. 3, 4 (2009).

Human research participants

Policy information about studies involving human research participants and Sex and Gender in Research.

Reporting on sex and gender	N/A
Population characteristics	N/A
Recruitment	N/A
Ethics oversight	N/A

Note that full information on the approval of the study protocol must also be provided in the manuscript.

Field-specific reporting

Please select the one below that is the best fit for your research. If you are not sure, read the appropriate sections before making your selection.

☒ Life sciences ☐ Behavioural & social sciences ☐ Ecological, evolutionary & environmental sciences

For a reference copy of the document with all sections, see nature.com/documents/nr-reporting-summary-flat.pdf

Life sciences study design

All studies must disclose on these points even when the disclosure is negative.

Sample size	No statistical sample size calculation was performed. Sample sizes were similar to or larger than previous publications in the field and sufficient for our claims:<br><br>- For stripe networks: Schaerli, Y. et al. Nat. Commun. 5, 4905 (2014), Santos-Moreno, J. et al. Nat. Commun. 11, 2746 (2020).<br>- For the oscillator: Niederholtmeyer, H. et al. eLife 4, e09771 (2015), Santos-Moreno, J. et al. Nat. Commun. 11, 2746 (2020).
Data exclusions	No data were excluded.
Replication	Experiments were performed using at least three biological replicates. All data were reliably replicated.
Randomization	Biological replicates were obtained from cultures inoculated from single colonies chosen randomly from agar plates. The samples were not randomized for microplate experiments since the position of samples within the plates and the order of the signal acquisition are not expected to affect the conclusion. Different induction conditions were always measured back to back for a given construct. Any covariate will affect all conditions equally hence rendering the effect not relevant.
Blinding	Blinding was not relevant since experiments did not involve any strain allocation to particular treatment groups and subsequent group comparison.

Reporting for specific materials, systems and methods

We require information from authors about some types of materials, experimental systems and methods used in many studies. Here, indicate whether each material, system or method listed is relevant to your study. If you are not sure if a list item applies to your research, read the appropriate section before selecting a response.

Materials & experimental systems

n/a	Involved in the study
☒	☐ Antibodies
☒	☐ Eukaryotic cell lines
☒	☐ Palaeontology and archaeology
☒	☐ Animals and other organisms
☒	☐ Clinical data
☒	☐ Dual use research of concern

Methods

n/a	Involved in the study
☒	☐ ChIP-seq
☒	☐ Flow cytometry
☒	☐ MRI-based neuroimaging
