# Supplementary material for: Robustness and innovation in synthetic genotype networks
Source: Nat Commun. 2023 Apr 28;14:2454. doi: 10.1038/s41467-023-38033-3 (PMC10147661; doi:10.1038/s41467-023-38033-3)
Supplement: Supplementary file 3 — Description of Additional Supplementary Files [file 41467_2023_38033_MOESM3_ESM.pdf]

Title: Supplementary Data 1

Description: Model parameters.

Title: Supplementary Data 2

Description: Annotated plasmid sequences.
